# Multiparametric MRI characterization of knee articular cartilage and subchondral bone shape in collegiate basketball players

**DOI:** 10.1002/jor.24851

**Published:** 2020-09-17

**Authors:** Kenneth T. Gao, Valentina Pedoia, Katherine A. Young, Feliks Kogan, Matthew F. Koff, Garry E. Gold, Hollis G. Potter, Sharmila Majumdar

**Affiliations:** ^1^ Department of Radiology and Biomedical Imaging University of California San Francisco California USA; ^2^ Department of Radiology Stanford University Stanford California USA; ^3^ Department of Radiology and Imaging Hospital for Special Surgery New York City New York USA

**Keywords:** basketball, bone shape, knee articular cartilage, magnetic resonance imaging, *T*_1ρ_‐ and *T*_2_‐relaxation

## Abstract

Magnetic resonance imaging (MRI) is commonly used to evaluate the morphology of the knee in athletes with high‐knee impact; however, complex repeated loading of the joint can lead to biochemical and structural degeneration that occurs before visible morphological changes. In this study, we utilized multiparametric quantitative MRI to compare morphology and composition of articular cartilage and subchondral bone shape between young athletes with high‐knee impact (basketball players; *n* = 40) and non‐knee impact (swimmers; *n* = 25). We implemented voxel‐based relaxometry to register all cases to a single reference space and performed a localized compositional analysis of *T*_1ρ_‐ and *T*_2_‐relaxation times on a voxel‐by‐voxel basis. Additionally, statistical shape modeling was employed to extract differences in subchondral bone shape between the two groups. Evaluation of cartilage composition demonstrated a significant prolongation of relaxation times in the medial femoral and tibial compartments and in the posterolateral femur of basketball players in comparison to relaxation times in the same cartilage compartments of swimmers. The compositional analysis also showed depth‐dependent differences with prolongation of the superficial layer in basketball players. For subchondral bone shape, three total modes were found to be significantly different between groups and related to the relative sizes of the tibial plateaus, intercondylar eminences, and the curvature and concavity of the patellar lateral facet. In summary, this study identified several characteristics associated with a high‐knee impact which may expand our understanding of local degenerative patterns in this population.

## 1 INTRODUCTION

The knee is vulnerable to articular cartilage degeneration and injury in jumping athletes who exert high compressive and shear forces during practice and competitive play.^1^, ^2^, ^3^, ^4^, ^5^ Imparting large loads to the articular cartilage is a known risk factor for chronic musculoskeletal conditions such as early‐onset osteoarthritis (OA)^1^ and pain.^2^ Accordingly, there is wide interest in studying associations between high‐knee impact sports and long‐term health of the knee joint.

Articular cartilage is of distinct concern due to its specialized function for distributing loads and its limited capacity for repair. Previous studies have used magnetic resonance imaging (MRI) to find that degenerative changes are consistently prevalent in knee cartilage of basketball players across all levels of competition.^3^, ^4^, ^5^ A 2005 study observed articular cartilage lesions on MRI in 47.5% of asymptomatic professional NBA players, with the majority of cartilage lesions found in the patellofemoral joint.^3^ A recent study by Pappas et al.,^4^ imaged 24 NCAA Division I collegiate basketball players and found increased abnormal findings (fat pad edema, patellar tendinopathy, articular cartilage, and meniscal injury) after one season of play in every knee imaged.^5^

Though the high prevalence of abnormal imaging findings in high‐knee impact athletes is well‐established, biochemical changes of macromolecules associated with cartilage degeneration occur before visible morphological changes.^6^, ^7^ Biomechanical stiffness of articular cartilage is provided by the collagen and proteoglycan (PG) organization and content, respectively, of the extracellular matrix (ECM). Damage to this macromolecular environment results in an increase of mobile water and a concomitant reduction in tissue stiffness. Compositional MRI techniques, such as *T*_1ρ_‐ and *T*_2_‐relaxation time mapping, can quantify such changes in cartilage matrix biochemistry.^8^, ^9^ *T*_1ρ_‐relaxation times reflect interactions between movement‐restricted water and surrounding large macromolecules and has been related to glycosaminoglycan (GAG) and PG content and early OA. Some studies demonstrated elevated *T*_1ρ_‐relaxation times with disruption of the ECM through decreased PG content via ex vivo enzymatic removal,^10^, ^11^ yet others have seen no relation between *T*_1ρ_ abnormalities and GAG.^12^, ^13^ While the mechanism is not yet fully understood, prolonged *T*_1ρ_ has associated with populations at risk of and living with OA.^14^, ^15^, ^16^ Meanwhile, *T*_2_ relaxation is associated with loss of collagen and disorganization of collagen fibrils.^16^ *T*_2_ is prolonged in the setting of the degeneration of articular cartilage.^11^ Newer methods permit acquisitions of *T*_1ρ_ and *T*_2_ in a single combined sequence and have been used to evaluate patients with anterior cruciate ligament injuries and those with OA,^16^, ^17^ but its use to investigate the status of knee cartilage health in young elite athletes is limited.

Quantitative analysis of *T*_1ρ_‐ and *T*_2_‐relaxation time maps are traditionally performed using region of interest (ROI)‐based approaches, which presents several challenges: (1) cartilage ROIs are often segmented manually or semi‐automatically and are prone to inter‐ and intrauser variation; (2) statistical analyses are performed based on the average *T*_1ρ_ or *T*_2_ value of all voxels within the ROIs, limiting the spatial assessment of relaxation times within the defined regions. Methods for segmentation have recently advanced to be less reliant on manual input. Advanced segmentation methods transform images from individual knees to a single reference template, allowing comparison of local spatial distribution between subjects on a voxel‐by‐voxel basis. This technique, voxel‐based relaxometry (VBR), has been shown to agree with ROI‐based analyses.^18^ Notably, it can be performed in a fully automated fashion and can provide local information and patterns of imaging markers in articular cartilage evaluation.^18^

Another component that plays a key role in the transmission of load across the knee joint is the geometric bone shape. Through skeletal homeostatic signal pathways,^19^ high‐intensity mechanical loading is associated with increased subchondral bone thickness and reduced bone resorption.^19^, ^20^, ^21^ Stimulation of these pathways occurs in an anatomic site‐specific manner depending on intensity and type of load. In turn, exercise‐induced variations in bone architecture influence biomechanics of the knee joint^22^, ^23^ and incidence rates of injury^24^ and OA.^25^ Due to frequent heavy loads exerted onto the knees of athletes in high‐knee impact sports, it is important to classify regional bone shapes in sports with low‐ and high‐knee impact.

Statistical shape modeling (SSM) has recently gained traction as an analytical method for modeling variation in surface geometry from imaging.^26^, ^27^ Varying algorithms have demonstrated submillimeter level matching precision, allowing for analysis of complex three‐dimensional (3D) shapes generated from medical imaging.^26^, ^27^, ^28^, ^29^

The purpose of this study was to use quantitative MRI techniques to characterize the articular cartilage and subchondral bone within the knee of two athletic groups: (1) a high‐knee impact group consisting of collegiate basketball players, and (2) a non‐knee impact group of collegiate swimmers. We hypothesized that the basketball players would demonstrate localized prolonged *T*_1ρ_‐ and *T*_2_‐relaxation times and bone shape differences as compared to the swimmers.

## 2 METHODS

### 2.1 Subject demographics

In this multicenter cross‐sectional study (Level 2), two cohorts of age‐matched NCAA collegiate‐level athletes were recruited for this study: 40 basketball players (22 female/18 male, 19.5 ± 1.5 years, body mass index [BMI] = 24.6 ± 5.6 kg/m^2^), and 25 swimmers (12 female/13 male, 19.0 ± 1.0 years, BMI = 25.4 ± 4.9 kg/m^2^). Participants were questioned about overall knee health and past history of competitive sports participation. Swimmers with a prior knee injury, pain, surgery, or participation in competitive jumping sports were excluded. Procedures were performed in accordance with the rules approved by the Institutional Review Boards of the three participating sites. All participants provided informed written consent.

### 2.2 MRI protocol

Imaging was performed using clinical 3T MRI (GE Healthcare) scanners with an 8‐channel T/R (Invivo) or an 18‐channel T/R knee coil (Quality Electrodynamics). Images were acquired before the subjects’ respective basketball and swimming competitive seasons. The single‐knee MRI protocol included a sagittal 2D fast spin‐echo (FSE) proton‐density (PD)‐weighted sequence, a sagittal intermediate‐weighted 3D FSE CUBE sequence, and a 3D‐sagittal combined *T*_1ρ_/*T*_2_ magnetization‐prepared angle‐modulated portioned *k*‐space spoiled gradient‐echo snapshots (MAPSS) research sequence.^17^ In the *T*_1ρ_ component of the MAPSS acquisition, time of spin‐lock (TSL) was set to 0/10/40/80 ms using RF pulse with a frequency of 500 Hz. Simultaneous *T*_2_ acquisition used echo time (TE) = 0/12.8/25.7/51.4 ms, sharing the first image with the first *T*_1ρ_ TSL. Additional acquisition parameters are listed in Table 1.

**Table 1:** MRI imaging acquisition parameters

	2D PD FSE	3D CUBE	3D MAPSS
Matrix	512 × 384	512 × 512	256 × 128
Field‐of‐view (cm)	16	16	14 or 16
Pixel bandwidth (Hz)	163	244	488
Slice thickness (mm)	3	0.7	4
Number of slices	30–45	145–210	24
Repetition time (ms)	5800	1200	5400
Echo time (ms)	40	27	0/12.8/25.7/51.4
Echo train length	14	35	1
Spin lock time (ms)	–	–	0/10/40/80
Flip angle	142°	90°	60°
ARC acceleration factor	–	Phase: 2.0	Phase: 2.0
		slice: 2.0	slice: 1.0
Approximate scan time (min:s)	4:30	6:30	9:40

Abbreviations: 3D, three‐dimensional; FSE, fast spin‐echo; MAPSS, magnetization‐prepared angle‐modulated portioned *k*‐space spoiled gradient‐echo snapshots; MRI, magnetic resonance imaging; PD, proton‐density.

To assess biases in quantitative measurements across the sites of acquisition, an identical phantom was imaged on all scanners. The phantom was constructed with two instances of three varying amounts of agarose to encompass a range of relaxation times and scanned with the *T*_1ρ_/*T*_2_ MAPSS sequence.^17^ The phantom acquisition was repeated two additional times at a single site to evaluate intrascanner variability. Coefficients of intrascanner variation ranged from 0.2% to 2.2%, while coefficients of interscanner variation ranged from 4.1% to 6.6%.^30^

### 2.3 Morphological characterization

A board‐certified musculoskeletal radiologist with 25 years of experience evaluated the MR images. Cartilage lesions were graded in a blinded fashion using the modified Noyes score, where Grade 0 classified cartilage with no lesions by PD‐weighted MRI, and Grades 1 and above indicated increased signal intensity or cartilage defects.

### 2.4 Voxel‐based relaxometry

Image postprocessing was performed using toolboxes implemented in MATLAB (MathWorks).

For compositional analysis, all cases with cartilage lesions (modified Noyes ≥ 1) in any compartment, identified by morphological characterization, were not considered to focus on prestructural abnormalities and early signs of biochemical change. Sagittal MAPSS images in all echoes were rigidly registered to the first TSL/TE = 0 of each case using the VTK CISG registration toolkit.^31^ Next, nonrigid registration to an atlas was then applied to all cases to morph the images to a common reference space. This was performed using Elastix,^32^ a medical imaging registration toolbox based on maximizing mutual information between the fixed and moving images. The resulting nonrigid transformations between the atlas and each TSL/TE = 0 cases were then applied to all other echoes/spin‐lock images. As all images were morphed to the same coordinate space, *T*_1ρ_ and *T*_2_ maps were calculated on a voxel‐by‐voxel basis using Levenberg–Marquardt mono‐exponential fitting.

### 2.5 ROI‐based relaxometry

Using a semiautomatic method based on edge‐detection,^33^ cartilage of the atlas was segmented into six compartments: lateral femoral condyle (LFC), medial femoral condyle (MFC), patella (PAT), trochlea (TRO), lateral tibia, and medial tibia (MT). The resulting masks were then applied to all morphed images.

A depth‐dependent ROI analysis was performed to evaluate variation between cartilage layers. Each of the above‐mentioned compartments was divided in half into a deep layer, closest to the subchondral bone, and a superficial layer, closest to the articular surface.

### 2.6 Statistical shape modeling

Segmentation of femur, tibia, and PAT bones were performed automatically using V‐Net,^34^ a fully convolutional neural network. Bones from 36 3D CUBE images were manually segmented for training, validation, and testing (26/6/4 split). Before training, all images were downsampled to 256 × 256 × 212 for computational efficiency. All training images were augmented with a random permutation of the following preprocessing techniques: additive gaussian noise, histogram matching, Gaussian filter, and affine transformation.

The V‐Net architecture implemented eight output channels in the first level, doubling at each of the subsequent three levels. One, two, and three convolutions were performed at each level, respectively, and three additional convolutional layers were added to the bottom level of the network. Dropout was implemented at 5% as a regularization penalty. The Dice coefficient was chosen as the loss function, with sigmoid activation applied, as well as the metric for evaluation. The model was trained for 24,000 iterations using a batch size of 1 and resulted in Dice coefficients of 0.98 ± 0.01, 0.98 ± 0.01, and 0.96 ± 0.01 (mean ± *SD*) for the femur, tibia, and PAT, respectively. The prediction algorithm was then applied to each case in the dataset.

The resulting segmentations were used to produce 3D triangulated meshes of the femur, tibia, and PAT bones using a Marching Cube algorithm.^35^ Next, with the bones in all cases being represented by clouds of points, each bone was nonrigidly registered using FOCUSR, as proposed by Lombaert et al.^36^ This method utilized spectral correspondence, which parametrizes vertex similarity using Laplacian eigen‐decomposition and then performs spectrum reordering via feature matching. The registered femurs, tibias, and PATs were described with 50,537, 33,210, and 8477 vertices, respectively.

Principal component analysis (PCA) was then performed to simplify the complexity of the surface data for interpretation. PCA transformed the vertex coordinates to orthonormal bases, where each principal component (PC) mode is uncorrelated and is ordered such that the first PC describes the direction of maximal bone shape variance and subsequent PCs are sorted in a descending manner. In consideration of the size of our dataset, 10 PC modes were sufficient to capture over 80% of the variance in each bone while still maintaining the physical interpretability of the surface models.

### 2.7 Statistical analysis

Morphological statistical analysis used a *χ*² test to assess the relationship of the prevalence of cartilage abnormalities between the two groups.

In the compositional analysis, summary statistics, including mean and *SD*, of *T*_1ρ_ and *T*_2_ times were computed and compared between basketball players and swimmers. This was computed for individual voxels in VBR, and in each cartilage compartment, and between cartilage layers in ROI‐based analysis. Group differences were assessed using a one‐way analysis of covariance (ANCOVA). Gender, BMI, and site of the acquisition were used as adjustment factors to control for confounding effects. A significance threshold was set at *p* < .05 (SPSS version 26.0; IBM).

Bone shape analysis involved evaluating PC values to determine if specific shapes were associated with the basketball or swim group. PCs that described shape differences related to the femur and tibial shafts were disregarded, due to variations in subject positioning during MRI acquisition and our specific interest in characterizing subchondral bone. An ANCOVA test, controlled for gender, BMI, and site of acquisition, determined statistical difference between groups. The physical representation of each mode was visualized in two ways: (1) average surface ± the displacement of each vertex by 3SDs, and (2) the average surface with the color mapping of the Euclidean norm at ±3SDs.

## 3 RESULTS

### 3.1 Morphological evaluation

The prevalence of cartilage abnormalities was significantly higher in the basketball group (*χ* ^2^ = 6.658, *p* < .01), occurring in 24.6% of knees of basketball players and 6.3% of knees of swimmers (Table 2). By compartment, this increase was significant in the LFC (*χ* ^2^ = 5.51, *p* < .05).

**Table 2:** Counts of abnormalities present and percentage of total imaged knees from MRI evaluation

		Basketball (65 knees)		Swim (48 knees)		*χ*	*p* Value
LFC	Noyes 0	58	(89.2%)	48	(100%)	5.51	Significant
	1	1	(1.5%)	0	(0%)		*p* < .05
	2	6	(9.2%)	0	(0%)		
MFC	Noyes 0	64	(98.5%)	47	(97.9%)	0.47	Insignificant
	1	0	(0%)	1	(2.1%)		*p* = .83
	2	1	(1.5%)	0	(0%)		
TRO	Noyes 0	63	(96.9%)	48	(100%)	1.50	Insignificant
	1	1	(1.5%)	0	(0%)		*p* = .22
	2	1	(1.5%)	0	(0%)		
PAT	Noyes 0	56	(86.2%)	46	(95.8%)	2.94	Insignificant
	1	2	(3.1%)	0	(0%)		*p* = .09
	2	6	(9.2%)	2	(4.2%)		
LT	Noyes 0	63	(96.9%)	48	(100%)	1.50	Insignificant
	1	0	(0%)	0	(0%)		*p* = .22
	2	2	(3.1%)	0	(0%)		
MT	Noyes 0	65	(100%)	48	(100%)	–	–
	1	0	(0%)	0	(0%)		
	2	0	(0%)	0	(0%)		
Total knees (Noyes ≥ 1)		16	(24.6%)	3	(6.3%)	6.66	Significant
						*p* < .01

*Note*: Cartilage was graded using a modified Noyes score (0 = normal; 1 = increased *T*_2_ in morphologically normal cartilage; 2 = partial‐thickness defect <50%). Grades above 2 were omitted due to absence in this dataset. A *χ* ^2^ test was performed to test the statistical frequency of the presence of cartilage abnormalities in each compartment and overall in each knee. The LFC demonstrated significant differences (*χ* = 5.51, *p* < .05), as well as across the combined compartments (*χ* = 6.66, *p* < .01). The test was not performed on the MT due to the absence of abnormalities.

Abbreviations: LFC, lateral femoral condyle; LT, lateral tibia; MFC, medial femoral condyle; MRI, magnetic resonance imaging; MT, medial tibia; PAT, patella; TRO, trochlea.

### 3.2 ROI analysis

**Figure 1:**
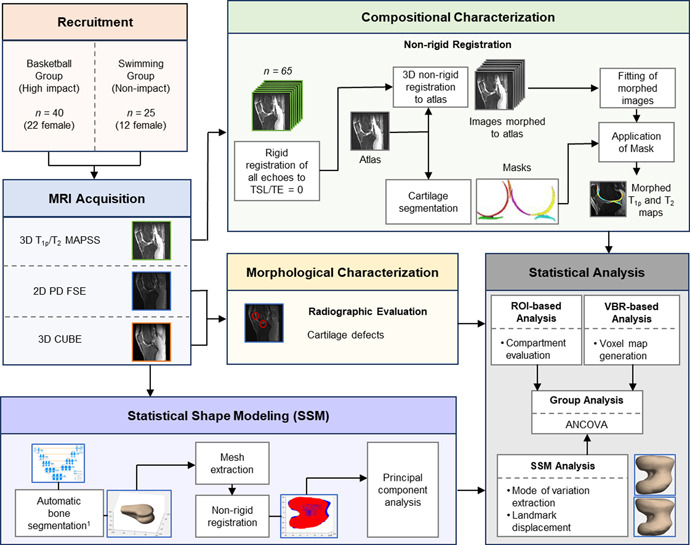
Schematic overview of study methodology. 3D, three‐dimensional; ANCOVA, analysis of covariance; FSE, fast spin‐echo; MAPSS, magnetization‐prepared angle‐modulated portioned *k*‐space spoiled gradient‐echo snapshots; MRI, magnetic resonance imaging; PD, proton‐density; ROI, region of interest; TSL, time of spin‐lock; VBR, voxel‐based relaxometry [Color figure can be viewed at wileyonlinelibrary.com]

Sixteen of sixty‐five basketball cases and three of forty‐eight swim cases included one or more defects in any cartilage compartment and were removed from ROI‐ and subsequent VBR‐based analysis to isolate differences in tissue composition. Mean *T*_1ρ_ and *T*_2_ values of the compartmentalized ROI‐based results ranged from 34.3 to 46.3 and 25.0 to 32.9* *ms, respectively (Figure 1).

**Figure 2:**
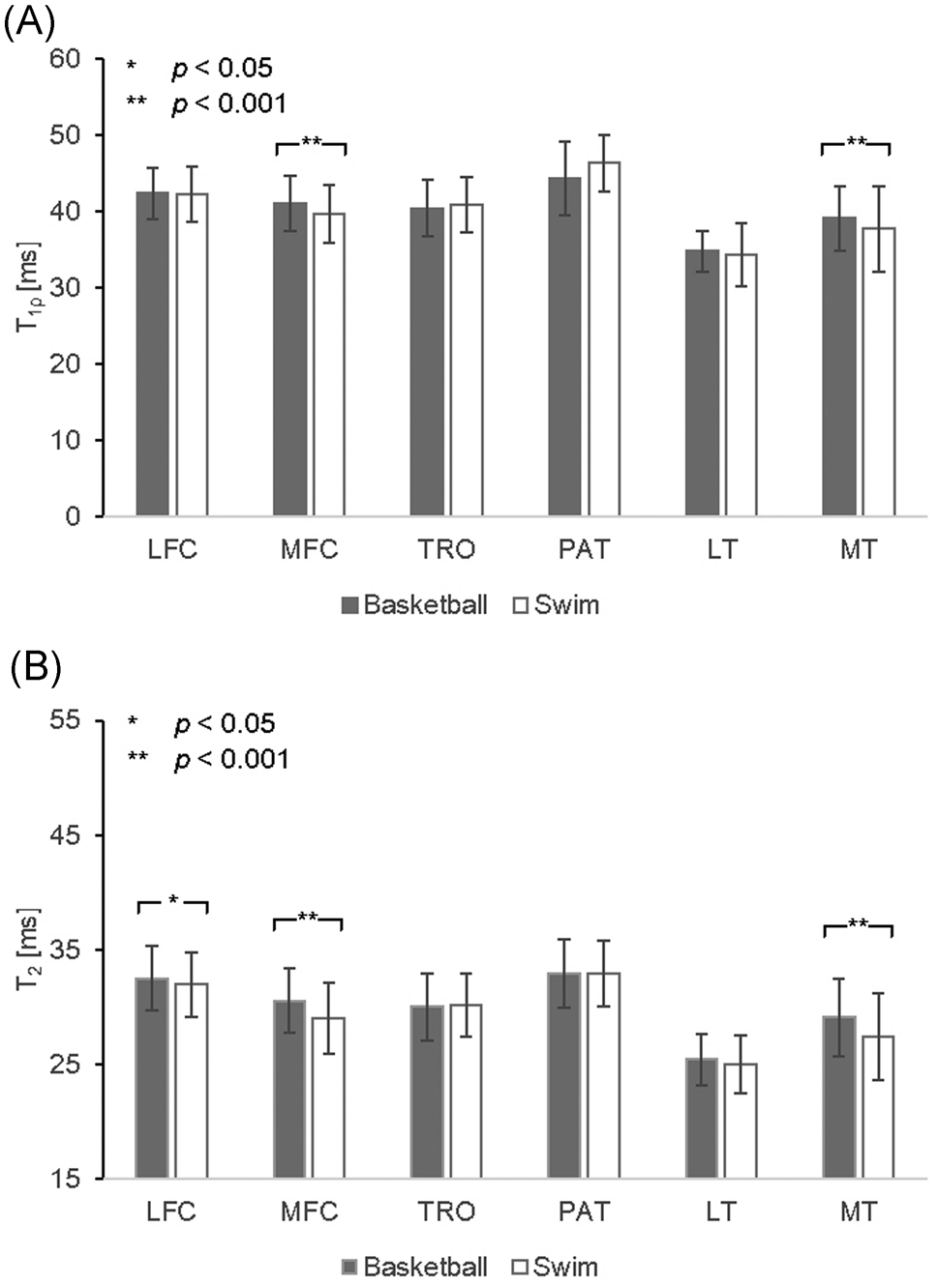
Results of the ROI‐based method for analysis of group differences in (A) *T*_1ρ_ and (B) *T*_2_ within cartilage compartments. Significant differences were found in the MFC and MT in both *T*_1ρ_ and *T*_2_, and additionally in the LFC in *T*_2_. LFC, lateral femoral condyle; LT, lateral tibia; MFC, medial femoral condyle; MT, medial tibia; PAT, patella; ROI, region of interest; TRO, trochlea

**Figure 3:**
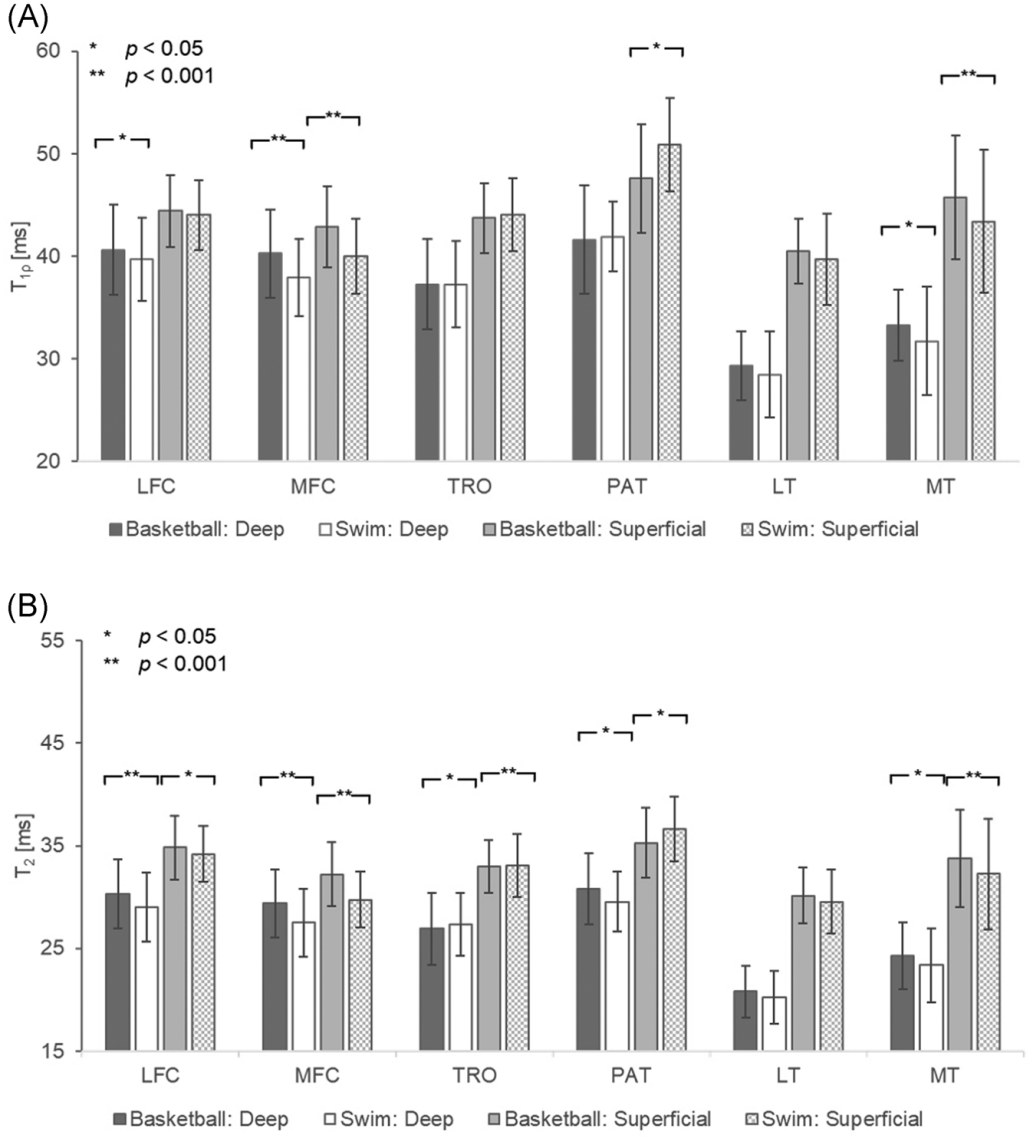
Depth‐dependent group comparison of mean (A) *T*_1ρ_ and (B) *T*_2_ relaxation times. Significant differences are labeled with *, while highly significant differences are labeled with **. In subcompartments with statistical significance, basketball players demonstrate prolonged relaxation times except in the superficial layer of the patellar and trochlear cartilage. LFC, lateral femoral condyle; LT, lateral tibia; MFC, medial femoral condyle; MT, medial tibia; PAT, patella; TRO, trochlea

The ROI‐based results demonstrate significant group differences in the medial compartment (Figure 2). The basketball group had significantly prolonged *T*_1ρ_ and *T*_2_ values in the MFC (*T*_1ρ_: 3.54% difference, *p* < .001 and *T*_2_: 3.63% difference, *p* < .001) and MT (*T*_1ρ_: 5.28% difference, *p* < .001 and *T*_2_: 6.04% difference, *p* < .001) compartments, as well as prolonged *T*_2_ values in the LFC compartment (*T*_2_: 1.72% difference, *p* < .05), though this lateral association was weaker. No significant differences were detected in the patellofemoral compartment (*T*_1ρ_: 4.30% difference, *p* = .53 and *T*_2_: 0.17% difference, *p* = .43) using the ROI‐based technique (Figure 3).

Cartilage compartments were further partitioned into a deep and superficial layer to evaluate variations in cartilage depth. When comparing these laminar features in each compartment, *T*_1ρ_ and *T*_2_ of the superficial layer were significantly prolonged (*p* < .001 in all cases). Group analysis demonstrated similar results to those in Figure 2 before partitioning, with statistically prolonged *T*_1ρ_ in the deep layer of the LFC (2.36% difference, *p* < .001), both layers of the MFC (deep: 6.05% difference, *p* < .001; superficial: 6.84%, *p* < .001) and MT (4.78% difference, *p* < .05; superficial: 5.21% difference, *p* < .001) in basketball players. *T*_2_ was similarly prolonged in basketball players for most cartilage compartments: LFC (deep: 4.29% difference, *p* < .001; superficial: 1.76% difference, *p* < .05), MFC (deep: 6.64% difference, *p* < .001; superficial: 7.94% difference, *p* < .001), PAT (deep: 4.09% difference, *p* < .05) and MT (deep: 3.97% difference, *p* < .05; superficial: 4.56% difference, *p* < .001). The only subcompartment where relaxation times of swimmers were higher than those of basketball players was the superficial layer of the patellar (*T*_1ρ_: 6.68% difference, *p* < .05; *T*_2_: 3.77% difference, *p* < .05), and trochlear cartilage (*T*_2_ deep: 1.52% difference, *p* < .05; *T*_2_ superficial: 0.20% difference, *p* < .001).

### 3.3 VBR analysis

Interpretation of the mean *T*_1ρ_ and *T*_2_ maps from VBR‐displayed prolongation near the trochlear groove and areas of shortening in the anterior and posterior regions of the tibiofemoral articulation.

**Figure 4:**
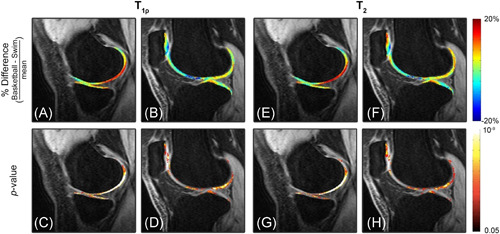
(A,B) Percentage difference map T¯basketball−T¯swimT¯basketball+T¯swim2, where $\overset{®}{T}$ is mean relaxation time, and (C,D) the respective *p*‐maps generated by VBR. Basketball players have significantly prolonged *T*_1ρ_ values in the posterior medial and lateral femoral condyles and tibial plateau. Meanwhile, there are laminar differences in the deep and superficial layers of the patellofemoral joint. Basketball players present with prolonged *T*_1ρ_ values in the deep layer and shorter *T*_1ρ_ values in the superficial layer, as compared to swimmers. (E–H) *T*_2_ differences and *p*‐maps show similar differences between groups. VBR, voxel‐based relaxometry [Color figure can be viewed at wileyonlinelibrary.com]

Comparison of the two groups demonstrated significant differences by sport, with basketball players generally with longer *T*_1ρ_ and *T*_2_ values, particularly in both femoral condyles (lateral: 12.63% average percentage difference, 42.7% significant voxels; medial: 3.48% average percentage difference, 29.2% significant voxels; Figure 4). The voxels that depicted significant prolongation were heavily focused on the posterolateral and posteromedial femur. Diffuse elevation was also noted in the anterocartilage.

Voxel‐based group analysis also revealed differences through the depth of the articular cartilage in the patellofemoral joint: basketball players had higher *T*_1ρ_ and *T*_2_ values in the deep layer of cartilage while swimmers had prolonged values in the superficial layer. This depth‐dependent distribution was not obviously evident in other regions evaluated and is of notable interest due to the vital role of the patellofemoral joint in the translation of weight.

### 3.4 Bone shape analysis

The femur, tibia, and PAT were each described in domains defined by 10 PC modes that maximize variation in shape. The amount of variability within the entire dataset, as represented by the PCs were 80.8%, 89.7%, and 82.5%, respectively.

**Figure 5:**
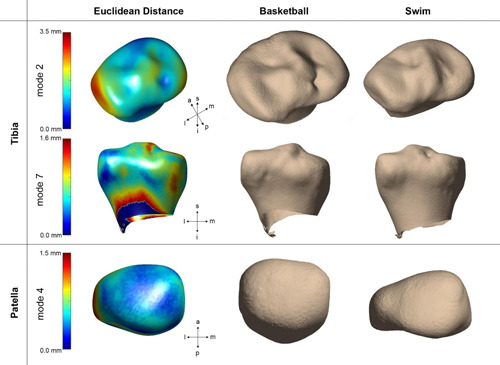
Bone shapes demonstrated significant group differences in the tibia and patella. In the first column, an average surface mesh is mapped in color by the Euclidean distance between the average surface mesh and +3*SD*. Models of basketball players and swimmers are shown in the second and third columns, respectively, represented by mean vertices displaced by ±3*SD*. (Top) Tibia mode 2 relates the size of the lateral plateau, (middle) tibia mode 7 primarily describes to relative heights of intercondylar spines, and (bottom) patella mode 4 show symmetry and curvature of medial and lateral facets [Color figure can be viewed at wileyonlinelibrary.com]

Among the 10 PCs of each bone, ANCOVA tests showed three total modes that were significantly different between groups (Figure 5): the second and seventh modes of the tibia, and the fourth mode of the PAT. Tibia mode 2 (*p* < .01, 22.0% of variance) describes the size of the lateral plateau relative to the medial plateau, particularly in the anterolateral aspect. Tibia mode 7 (*p* < .05, 1.87% of variance) represents the relative heights of the intercondylar eminence. PAT mode 4 (*p* < .01, 4.24% of variance) is related to the curvature and convexity of the lateral articular facet. Variance in this mode also seemed to be connected to patellar symmetry. As the lateral facet extended, in relation to the medial facet, it demonstrated increased convexity.

## 4 DISCUSSION

This multicenter study used multiparametric MRI to extensively characterize the articular cartilage and bone shape of knees of basketball players (high‐knee impact) and swimmers (no knee‐impact). We demonstrated significant group differences using morphological evaluation, compositional evaluation through a traditional ROI‐based and fully automatic VBR‐based techniques, and statistical subchondral bone shape comparison in the femur, tibia, and PAT.

Imaging abnormalities in the articular cartilage of basketball players has been a heavily studied area.^1^, ^2^, ^3^, ^4^, ^5^ Frequent jumping, running, pivoting/cutting motions applies a heavy mechanical load to the cartilage. The prevalence of morphological cartilage defects found in this study relates well to past findings in imaging studies of professional basketball players.^3^, ^5^ Distribution of defects across the cartilage compartments was also consistent, with the remarkably high pervasiveness of findings in the patellar cartilage, followed by the trochlear and femoral cartilage.

MRI studies of knee cartilage composition in basketball players are much more limited. In this study, we identified and removed knees with morphological abnormalities from analysis to highlight key differences in biochemistry between groups. Classic ROI segmentation and analysis of *T*_1ρ_ and *T*_2_ led to findings of significant group differences in the medial compartments. Recent in vivo experiments of the compartmental strain of the tibia show increased strain on the medial side of the tibial plateau with increased normalized walking speed, but not for the lateral side.^37^ Additionally, medial compartment OA is the most common form of OA.^38^, ^39^ Our ROI‐based analysis captured a pattern representative of this asymmetry; however, this method was not effective in finding local findings in other compartments. The traditional ROI‐based analysis detected no differences between groups for patellar cartilage, despite previous research indicating the prevalence of imaging findings in this compartment.^2^, ^5^

Overall, the VBR analysis was more sensitive to local differences. The prevalence of significant *T*_1ρ_ and *T*_2_ prolongation in the medial femur and MT was consistent with results found in ROI analysis and was characterized by a dominance of higher values in the posterior cartilage of the basketball group. Depth dependent differences were detected, as well. Basketball players demonstrated higher values in the superficial layer of the medial cartilages, as seen in both VBR and depth‐dependent ROI results. However, an opposite pattern was displayed on the lateral side where diffuse patches of higher relaxation times are evident in the deep layer. The superficial layer of cartilage is composed primarily of type II and IX collagen, aligned parallel to the surface to protect the deep layer from shear stress, while the deep layer contains higher PG content and collagen aligned perpendicular to the surface to resist compressive forces.^40^ The VBR results may suggest cartilage degeneration in areas of prolonged *T*_1ρ_ and *T*_2_. We speculate, these differences could be attributed to the complexity of joint loading, and the differences in mechanical loading between the two sports: the basketball group experiences relatively more compressive loading on the lateral side, possibly from pivoting/cutting motions and high magnitude jump‐landing. Similarly, the dichotomy of relaxation patterns in the patellar cartilage could possibly be due to its role in facilitating extension during jumping and squatting. Conversely, swimmers use high frequency, low‐magnitude flexion/extension movements, which may exert higher shearing and tensile forces on the superficial layer of the PAT and TRO. Clearly, further experiments are warranted in support of these mechanistic hypothesis.

External loading is known to influence subchondral bone shape and thickness via bone remodeling.^21^, ^22^ While our SSM results demonstrated no differences in femur shape, the significant modes of the tibia are especially relevant in controlling the biomechanics of the tibiofemoral joint. We found more symmetry between lateral and medial plateaus in basketball players as compared to swimmers. Functionally, the lateral plateau is convex in shape and performs translational motion to the concave medial plateau. The anterolateral plateau, specifically, experiences tibial subluxation during knee flexion, indicating tibiofemoral internal rotation.^41^ A high degree of rotation due to pivoting/cutting in basketball may contribute to the symmetry seen in the lateral plateau shape. Similarly, tibia mode 7 shows higher prominence of the medial spine in basketball players. With its physical connection to the anterior cruciate ligament and its proximity to the medial meniscus, a vital tissue in shock absorption, the asymmetric heights could be explained by increased mechanical loading and subsequent bone remodeling. The size of the tibial plateau^42^ and heights of the intercondylar eminence have been positively correlated with the prevalence of tibial osteophytes^43^ and OA.^44^ Therefore, tibia shape may be an important consideration in identifying the progression of knee kinematics, degeneration, and risk of injury in young athletes.

In regard to PAT shape, there was significant variation in the lateral facet between groups. The representative PAT of basketball players was more symmetric with a concave lateral facet, whereas that of swimmers was elongated and convex. Using Wiberg shape classification,^45^ the patellar shape of basketball players can be categorized as type I, showing congruency and concavity on both facets of the PAT. Meanwhile, the nonimpact group shows similarity to type III, with a convex and posterior‐sloping medial facet much smaller than the lateral facet. It is unclear how this shape is associated with swimmers, as there may be hidden confounding factors that were not accounted for in this study.

In summary, we identified several characteristics associated with high‐knee impact athletes, including prolonged *T*_1ρ_ and *T*_2_ relaxation in cartilage compartments and local depth‐dependent differences, as well as bone shape variations in the tibia and PAT. This study had several limitations. First, the morphological evaluation was performed by a single senior musculoskeletal radiologist. Second, the voxels generated with the 3D MAPSS sequence were large (0.6 mm × 1.2 mm × 4 mm) as compared to the cartilage thickness, a known intrinsic MRI limitation when balancing the factors of scan time, anatomic coverage, and voxel size. Even with the possible influence of partial volume artifacts, our results show significant statistical results sensitive to local distributions. This is particularly evident in the PAT, where cartilage is thicker. Finally, the playing season training regimen of the basketball players and swimmers was not incorporated into the current analysis. Each sport has different training regimens which may influence the results of the study in addition to the time of play and practice. Further evaluation of this dataset may incorporate the effects of position played, for basketball, or primary stroke, for swim. It is worthwhile to note that the findings of this study do not establish causation between play and *T*_1ρ_ or *T*_2_ prolongation or bone shape. This cross‐sectional study evaluates absolute quantitative measures at a single time point; future evaluations will incorporate the effect of one season of play and longitudinal changes in these populations to further establish our findings. The relationships described in the current study provide a comprehensive characterization of the knees of young athletes with considerably different loading patterns, derived from imaging alone.

## CONFLICT OF INTERESTS

The authors declare that there are no conflict of interests.

## AUTHOR CONTRIBUTIONS

Kenneth T. Gao, Valentina Pedoia, Matthew F. Koff, Garry E. Gold, Hollis G. Potter, Sharmila Majumdar contributed to research design. Katherine A. Young, Feliks Kogan­, and Matthew F. Koff acquired the data. Kenneth T. Gao, Valentina Pedoia, and Sharmila Majumdar performed the experiments. Kenneth T. Gao, Valentina Pedoia, Feliks Kogan­, and Matthew F. Kof, Hollis G. Potter, and Sharmila Majumdar contributed to the interpretation of the results. All authors provided critical feedback and approved the final submitted manuscript.
